# Recurrent obstructive uropathy secondary to pyometrocolpos in an 8-month-old infant: a case report

**DOI:** 10.1186/s13256-020-02543-1

**Published:** 2020-12-07

**Authors:** Richard Kiritta, Georgina Balyorugulu, Maimuna Ahmed, Frank Christopher, Andrea Solnes Miltenburg, Albert Kihunrwa

**Affiliations:** 1 Department of Obstetrics and Gynecology, Bugando Medical Centre, Catholic University of Health and Allied Sciences, Mwanza, Tanzania; 2 Department of Pediatrics, Bugando Medical Centre, Catholic University of Health and Allied Sciences, Mwanza, Tanzania; 3 Department of Obstetrics and Gynecology, Akershus University Hospital, Lorenskog, Norway

**Keywords:** Pyometrocolpos, Pyometra, *Escherichia coli*, Abdominal mass, Urogenital malformation, Case report

## Abstract

**Background:**

Pyometrocolpos is accumulation of infected fluid in the uterus and vagina. It is rare in children, mostly seen after menarche as a result of obstructive congenital genital malformation that impairs free drainage of the uterine secretions. In a child, it may present as an acute illness that necessitates urgent and appropriate management and treatment of the underlying cause, which can be a challenge in a resource-limited setting.

**Case presentation:**

We report a case of pyometrocolpos in an 8-month-old African infant who presented with fever, vomiting, decreased urine output, and abdominal distension of 12 days’ duration. An abdominal examination revealed a subumbilical midline incision scar and a midline lower abdominal mass. She appeared to have presented at the emergency department with similar complaints 2 months earlier and had been diagnosed with pyometra, which was managed by emergency laparotomy for pus drainage, and she was kept on antibiotics. Recovery was established after 10 days of admission, and the patient was discharged to home. Her symptoms reappeared 2 months after the first presentation. Her blood work showed significant leukocytosis with neutrophilia, and abdominal ultrasound depicted bilateral hydronephrosis with hydroureters and a fluid-filled uterus. Examination under anesthesia in the operating theater revealed normal-looking female genitalia with a cribriform hymen, beneath which lied a transverse vaginal septum. Foul-smelling pus was aspirated through the septum, and septectomy was performed to allow 350 ml of pus to drain. A pus sample was sent for culture and sensitivity, and *Escherichia coli* sensitive to ceftriaxone and gentamicin was isolated.

**Conclusion:**

Pyometrocolpos is rare in childhood but should be suspected in a girl presenting with a midline lower abdominal mass accompanied with urinary obstructive symptoms associated with fever and gastrointestinal symptoms. *Escherichia coli* seems to be the most probable offending organism, but pus culture is crucial for antibiotic stewardship in proper management of the infection. Definitive treatment should focus on correcting the obstructive anatomical congenital deformity that caused the obstruction in order to avoid recurrence.

## Introduction

Pyometrocolpos is defined as an infection of fluid within the uterus and vagina, which is usually a consequence of genital urinary malformations that impair free uterine drainage. It rarely manifests in children, leading to delays in its diagnosis and treatment. We describe what we believe is the first reported case of pyometrocolpos in a child in northern Tanzania and the second case in all of Tanzania. Pyometrocolpos was first reported in Zanzibar in 1948. Because of its rarity coupled with limited diagnostic capabilities in a resource-limited setting such as Tanzania, pyometrocolpos in an infant poses a great challenge in both diagnosis and management, as reflected by a recurrence of the condition as seen in this case. We describe the clinical presentation, diagnostic modalities, and management challenges and outcome of pyometrocolpos in an infant.

## Case presentation

We present a case of an 8-month-old African infant readmitted at our facility 2 months after laparotomy. She presented with abdominal distension of gradual onset for 12 days, accompanied with difficulty in passing urine and stool and low-grade fever. Two months earlier, she had presented with similar symptoms, at which time a diagnosis of obstructive uropathy due to pyometra had been made. This was then managed by an emergency laparotomy and drainage of 400 ml of pus from the uterus and postoperative treatment with antibiotics. In her current admission, she had nonprojectile vomiting that was nonbilious, nonbloody, and of food content.

Clinical assessment revealed she was febrile (38 °C) with anthropometric measurements indicating moderate acute malnutrition. Abdominal examination revealed a healed subumbilical median incision scar with a mobile, regularly shaped, smooth margin suprapubic mass extending up to the level of the umbilicus. The girl’s complete blood count showed leukocytosis of 26.69 × 10^9^ cells/L with a predominance of neutrophils at 15.83 × 10^9^cells/L (59.3%) and a hemoglobin count of 14.5 g/dl.

**Fig. 1:**
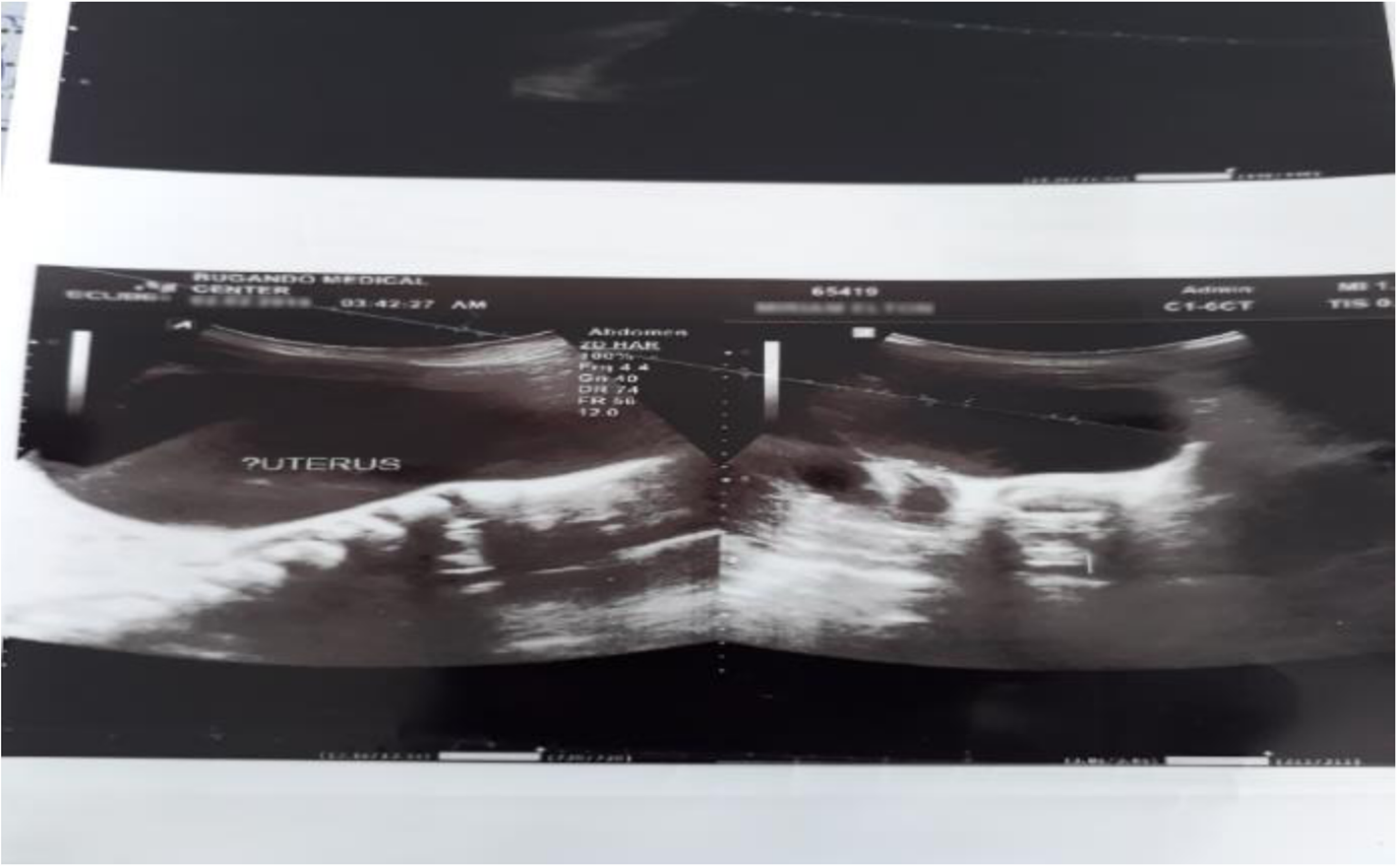
Midline longitudinal view of the pelvis showing fluid-filled mass in the shape of the uterus with echogenic area representing thicker fluid with bladder displaced to the left of a hydrolyzed kidney

An urgent abdominal pelvic ultrasound revealed a fluid-filled mass in the shape of the uterus with an echogenic area representing thicker fluid in the uterine cavity. The urinary bladder was displaced to the left, and the ureter and renal calyces were dilated, suggesting bilateral obstructive nephropathy secondary to a grossly distended uterine cavity with pyometra (Fig. 1).

**Fig. 2:**
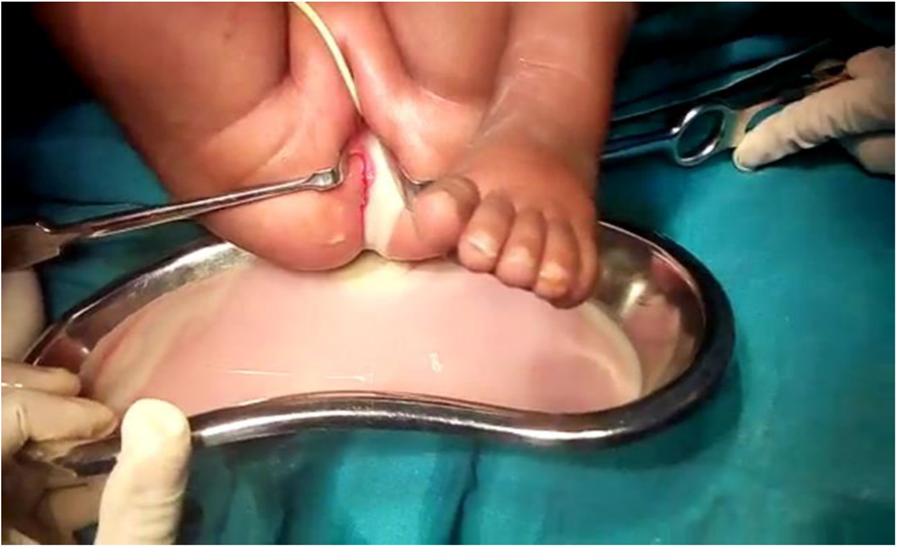
After hymenectomy and septectomy, 350 ml of pus was drained

The infant was reviewed by a gynecologist, who ordered an estrogen assay and renal function tests, the results of which all appeared to be within normal ranges for age. In order to perform a proper assessment of the underlying malformations, clinical examination was continued with the patient under general anesthesia in the operating theater. In the operating theater with the patient under general anesthesia and in lithotomy position, vaginal examination revealed a cribriform hymen beneath which a midvaginal transverse septum could be seen. A 5-ml syringe was carefully inserted at the middle of the septum, and a dense yellowish fluid was aspirated. Hymenectomy had to be performed to allow septectomy, and about 350 ml of pus were drained (Fig. 2). A pus sample was taken for bacterial culture and sensitivity analysis, which revealed *Escherichia coli* sensitive to ceftriaxone and gentamicin.

Uterine lavage was performed, and a size 16 three-way catheter was introduced through the excised septum site for continuous drainage and to maintain patency along the excised margins for 10 days while the infant was in the ward.

After 10 days, the drain (catheter) was removed, and the patient was observed for additional days before discharge. Six weeks after septectomy, she was seen for a follow-up appointment at the outpatient gynecology clinic. Abdominal pelvic ultrasound was performed, which revealed a normal empty uterus.

Physical examination revealed that the septum patency was maintained, and the patient’s nutritional status had improved significantly. She was then discharged from the gynecology clinic.

## Discussion

Pyometrocolpos refers to accumulation of infected fluid in the vagina and uterus. Developmental anomalies of the müllerian duct, in particular disorders of vertical (transverse) fusion resulting from abnormal canalization of the vaginal plate or failure of the uterovaginal primordium and the sinovaginal bulbs to fuse, can result transverse vaginal septum (TVS), an imperforate hymen, and in extreme cases a vaginal atresia [1]. These congenital malformations may cause secretions from the reproductive tract to accumulate in the vagina and uterus and become infected [2]. Obstructive vaginal malformations are usually asymptomatic in childhood or rarely become symptomatic due to mucocolpos with stimulation from maternal estradiol. Most cases become symptomatic after menarche following accumulation of menstrual blood proximal to the site of obstruction [3].

Our patient’s case is among the few occurring in childhood, with this particular infant undergoing surgery twice as a result of obstructive uropathy secondary to a recurrent pyometrocolpos due to a midvaginal transverse septum that was not corrected at the initial presentation. Similar to most reported cases, the clinical presentation in our patient’s case involved abdominal distension accompanied by both urinary and bowel obstructive symptoms together with gastrointestinal disturbances such as vomiting and irritability [4].

The presence of a suprapubic mass identified as a distended uterus filled with fluid and obstructive uropathy was made through clinical examination and confirmed by ultrasound, similarly to other cases. However, unlike some other cases in which computed tomography and magnetic resonance imaging were further ordered to confirm the diagnosis and evaluate underlying anatomical anomalies, in our patient’s case, financial constraints and the urgency for intervention resulted in immediate surgery without further imaging studies as is noted in an almost similar case in Ethiopia [5]. In resource-limited settings, a clinician might have to rely solely on clinical presentation and ultrasonographic findings for diagnosis of pyometrocolpos in children.

Proper management apart from the surgical intervention requires identification of the organism that caused the infection that resulted in pus formation. The bacterium that has been most associated with pyometrocolpos as obtained from either pus or blood sample cultures is *Escherichia coli.* Of 12 cases reported, only 33% yielded bacterial growth: 3 cases (75%) identified *E. coli* as the causative organism, and *Pseudomonas* was detected in 1 case [6]. In our patient’s case, *E. coli* was identified, which was sensitive to ceftriaxone and gentamicin.

Little is known about the cause of pyometra and pyometrocolpos in infants due to the rarity of the condition. Some authors hypothesize that hypoestrogenized endometrium in infants is thinner and less resistant to infection than a thicker estrogenized endometrium. A hypoestrogenized endometrium combined with an obstruction to uterine drainage may lead to hydrometra or hydrometrocolpos and consequently pyometra or pyometrocolpos, respectively [6]. The infant in our case had estrogen levels within the normal range for her age; hence, the mechanical obstruction to uterine drainage was the main cause of pyometrocolpos. However, as in other reported cases [2, 7], malnutrition appears to be a risk factor for the development of pyometrocolpos in our patient’s case.

Treatment of pyometrocolpos should entail identification of the underlying obstructive cause that predisposes patients to accumulation of fluid in the uterus and vagina; in our patient, the midvaginal transverse septum was identified. The failure to remove this mechanical obstruction during the first encounter led to persistent blockage, which resulted in reemergence of the condition 2 months later. Similar observation was noted in a case that involved a 10-month-old infant in whom the first surgery was performed to drain the uterus and in turn temporarily relieved the patient of the obstructive symptoms; however, a second surgical intervention was needed to insert a drain for continuous drainage of the uterine cavity [6].

TVS can occur at nearly all levels in the vagina (superior, middle, and inferior), with 46% superior, 40% middle, and 14% inferior [8]. Unlike the superior vaginal septum, which might require laparotomy to excise, middle and inferior septa can be approached vaginally, and, because of their thinness in infants and children, multiple radical incisions with anastomosis of upper and lower vaginal mucosa followed by application of a silicone vaginal form were the modality of treatment in a related case [5]. In our patient’s case, after examination with the patient under anesthesia, a vaginal approach was preferred because of ease of access to a midvaginal septum. A vaginal form is not available in these settings, and therefore a size 16 urinary catheter was inserted instead. This catheter helped to prevent fusion of septal incision edges, and hence it offers an alternative in areas where vaginal forms are not available.

## Conclusion

The diagnosis of pyometrocolpos, even though it is a rare condition in childhood, should be suspected in a girl presenting with urinary obstructive symptoms with a midline abdominal mass associated with fever and gastrointestinal complaints. Diagnosis in low-resource settings often relies solely on clinical presentation, physical examination, and ultrasound imaging. Management should include ruling out and correcting the congenital genital abnormality, which is crucial in ensuring that the surgical intervention is focused and curative and prevents recurrence.

## Patient’s perspective

The care provided was timely with full explanation of the diagnosis and prognosis and with a follow-up plan explained. Mother’s statement: “It is was a relief for my family to learn of my child’s condition and final diagnosis and treatment. We are thankful for the treatment and management.”
